# Genomic Landscape, Targeted Therapies, and Mechanisms of Resistance in Molecularly Selected Metastatic Colorectal Cancer Patients

**DOI:** 10.3390/genes17040460

**Published:** 2026-04-15

**Authors:** Patricia Garcia Pastor, Nadia Saoudi González, Francesc Salva, Javier Ros, Iosune Baraibar, Marta Rodríguez Castells, Clara Salva de Torres, Ariadna García, Adriana Alcaraz, Caterina Vaghi, Josep Tabernero, Elena Elez

**Affiliations:** 1 Medical Oncology Department, Vall d’Hebron University Hospital, 08035 Barcelona, Spain; patricia.garciapastor@vallhebron.cat (P.G.P.); fsalva@vhio.net (F.S.); fjros@vhio.net (J.R.); ibaraibar@vhio.net (I.B.); martarodriguez@vhio.net (M.R.C.); clarasalva@vhio.net (C.S.d.T.); jtabernero@vhio.net (J.T.); meelez@vhio.net (E.E.); 2 Colorectal Cancer Group, Digestive Tumors Group, Vall d’Hebron Institute of Oncology, VHIO, 08035 Barcelona, Spain; agarcia@vhio.net (A.G.); adrianaalcaraz@vhio.net (A.A.); caterinavaghi@vhio.net (C.V.); 3 Department of Hematology, Oncology, and Molecular Medicine, Niguarda Cancer Center, Grande Ospedale Metropolitano Niguarda, Piazza Ospedale Maggiore, 3, 20162 Milan, Italy

**Keywords:** metastatic colorectal cancer, targeted therapy, circulating tumor DNA, precision oncology

## Abstract

Metastatic colorectal cancer (mCRC) remains one of the leading causes of cancer-related mortality worldwide despite substantial therapeutic improvements over the past two decades. Advances in the understanding of colorectal tumor biology and oncogenic signaling have enabled the development of biomarker-guided therapies targeting alterations in *EGFR*, *BRAFV600E*, *KRAS* mutations and *HER2* amplifications, improving outcomes in selected patient populations. Nevertheless, the emergence of both intrinsic and acquired resistance mechanisms continues to limit the durability of these responses. Resistance to targeted therapies in mCRC arises through multiple, often convergent mechanisms, including activation of compensatory signaling pathways, pre-existing genomic heterogeneity, and therapy-driven clonal selection. The integration of molecular profiling into clinical decision-making is essential to guide treatment selection and optimize therapeutic sequencing, ultimately enabling progress in precision oncology. Advances in genomic technologies, particularly circulating tumor DNA (ctDNA) analysis, have allowed longitudinal monitoring of tumor evolution, providing important insights into the mechanisms underlying resistance to targeted therapies. The aim of this review is to summarize the genomic landscape of mCRC and discuss current targeted therapeutic strategies in molecularly defined subgroups, with a particular focus on the mechanisms driving primary and acquired resistance.

## 1. Introduction

Colorectal cancer (CRC) remains the third most commonly diagnosed malignancy worldwide and the second leading cause of cancer-related death, despite substantial therapeutic advances over the past decades and declining incidence in average-age populations in high-income countries [1,2]. Approximately 20% of patients present with metastatic disease at diagnosis, while an additional 30–50% of those initially diagnosed with localized tumors eventually develop metastatic relapse [3,4]. In addition, the incidence rates in people younger than 50 years have been increasing for the last 20 years, potentially driven by early-life environmental and lifestyle exposures, highlighting the need for deeper biological insight and more effective therapeutic approaches [5].

The pathogenesis of CRC is widely considered a multistep process in which normal colonic epithelium progressively evolves through precursor lesions into invasive carcinoma. Advances in histopathological and molecular characterization of colorectal tumorigenesis have led to the identification of distinct pathways of CRC development. It develops through three major molecular pathways: chromosomal instability (CIN), which accounts for approximately 65–70% of cases and is characterized by widespread copy number alterations; microsatellite instability (MSI-H), present in around 15% of cases and associated with defects in the DNA mismatch repair system; and the serrated pathway, observed in 15–30% of tumors and frequently linked to CpG island methylator phenotype (CIMP). These pathways are associated with distinct genomic and epigenetic alterations that contribute to tumor initiation and progression. Across these pathways, tumorigenesis is driven by disruption of key signaling networks, including the MAPK, Wnt/β-catenin, TGF-β, and PI3K-AKT pathways, which regulate cell proliferation, survival, and metastatic dissemination [6]. Deregulation of these cascades is primarily caused by recurrent genetic alterations in critical driver genes such as *APC* (80%), *TP53* (60%), *KRAS* (40%), *PIK3CA* (15–20%), and *BRAF* (8–12%), which not only promote colorectal carcinogenesis but also create therapeutic vulnerabilities that can be targeted by precision-based strategies [7,8].

Beyond the genomic alterations, CRC biology is also profoundly shaped by immune surveillance and transcriptional programs governing tumor-microenvironment interactions. Gene expression-based consensus molecular subtypes (CMS1-CMS4) capture dimensions of CRC heterogeneity that are not fully explained by DNA mutations alone, providing a deeper understanding. CMS further refine CRC classification into four biologically distinct groups: CMS1 (MSI-immune), characterized by hypermutation and strong immune activation; CMS2 (canonical), marked by *WNT* and *MYC* pathway activation; CMS3 (metabolic), associated with metabolic dysregulation and frequent *KRAS* mutations; and CMS4 (mesenchymal), defined by stromal infiltration, TGF-β activation, and poor prognosis [9]. As tumors expand, they develop a microenvironment characterized by severe hypoxia and acidosis, conditions that have been recognized as a hallmark of tumor progression. These hypoxic conditions induce the expression of hypoxia-inducible factors 1 and 2 (HIF-1 and HIF-2), which, in turn, upregulate various growth and proangiogenic factors, representing another hallmark of colon cancer that has also been targeted by antiangiogenics such as bevacizumab or aflibercept [6,9].

**Figure 1:**
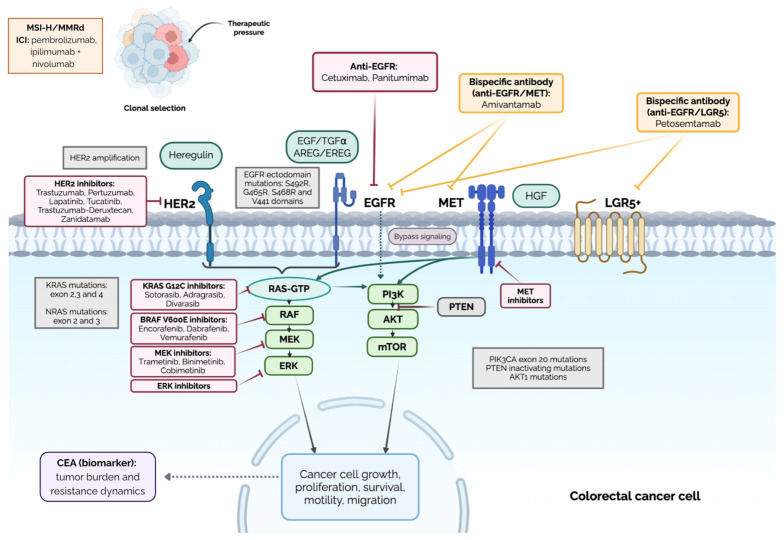
The dynamic landscape of genomic and non-genomic determinants of resistance and therapeutic targeted strategies in mCRC. Resistance mechanisms include activating mutations in key oncogenic pathways (MAPK and PI3K-AKT), receptor alterations (EGFR, HER2, MET), bypass signaling, and clonal evolution. To overcome these, several precision strategies are employed: anti-EGFR agents, which are being optimized through novel epitope antibodies or bispecifics to bypass receptor mutations. Targeting pathway reactivation involves BRAF inhibitors, MEK inhibitors, and KRAS G12Cspecific inhibitors. Furthermore, HER2-targeted strategies (tucatinib, trastuzumab-deruxtecan, zanidatamab) and MET inhibitors are used to block bypass signaling [8,12,13,14]. Abbreviations: MSI-H/MMRd: microsatellite instability/mismatch repair deficiency, ICI: immune-checkpoint inhibitors.

Building on these molecular insights, the therapeutic landscape of metastatic CRC has progressively shifted toward biomarker-driven strategies. The identification of actionable alterations, including *RAS* and *BRAF* mutations, *HER2* amplification, and *NTRK* fusions, has enabled the development of targeted agents that have significantly improved outcomes in selected patient subsets. Anti-EGFR antibodies, BRAF-directed combinations, and HER2-targeted therapies exemplify how molecular stratification has translated into clinically meaningful benefit. Despite these advances, responses to targeted therapies are often incomplete and rarely durable. Both primary (intrinsic) and acquired resistance inevitably emerge, limiting long-term efficacy. Resistance mechanisms in CRC are highly heterogeneous and frequently convergent, involving pre-existing subclonal alterations, activation of compensatory signaling pathways, feedback loop dysregulation, and therapy-driven clonal selection. Additional molecular and clinical factors contribute to therapeutic resistance. MSI-H tumors, although highly responsive to immune checkpoint inhibitors, may develop resistance through immune evasion mechanisms such as impaired antigen presentation or alterations in interferon signaling. *PIK3CA* mutations activate the PI3K-AKT pathway and have been associated with resistance to anti-EGFR therapies through downstream signaling reactivation. Carcinoembryonic antigen (CEA), while primarily used as a clinical biomarker, reflects tumor burden and biological aggressiveness, indirectly influencing treatment response and resistance dynamics. Tumor heterogeneity, whether spatial, temporal, or molecular, further complicates treatment, allowing resistant clones to expand under therapeutic pressure [10,11]. Figure 1 summarizes the main mechanisms underlying resistance to targeted therapies in mCRC.

This review synthesizes the current understanding of genomic resistance, focusing on mechanisms of primary and acquired resistance and highlighting future directions towards a more precise and dynamic management of mCRC.

## 2. EGFR Inhibitors in *RAS/BRAF*wt mCRC Patients

The epidermal growth factor receptor (EGFR) pathway was among the earliest molecular targets successfully exploited in the treatment of CRC. The EGFR (or ERBB1) belongs to the ERBB/HER protein family, a group of transmembrane tyrosine kinase receptors. EGFR is activated upon binding of several ligands, including epidermal growth factor (EGF), transforming growth factor α (TGFα), amphiregulin, epiregulin, and heparin-binding EGF-like growth factor. Ligand binding to the extracellular domain promotes receptor homo- or heterodimerization, triggering activation of downstream signaling cascades. The principal pathways involved include the RAS-RAF-MEK-MAPK (also called ERK) and PI3K-AKT-mTOR pathways, which regulate cellular proliferation, survival, migration, and differentiation [15,16].

Two major classes of EGFR-targeted agents have been developed: monoclonal antibodies directed against the extracellular receptor domain, and small-molecule tyrosine kinase inhibitors targeting the intracellular kinase domain. Among these agents, the monoclonal antibodies cetuximab and panitumumab have been approved in combination with chemotherapy in *RAS*/*BRAF* wild-type (wt) mCRC [17]. Panitumumab is a fully human IgG2 monoclonal antibody directed against EGFR. Cetuximab, in contrast, is a chimeric IgG1 monoclonal antibody, which explains its higher potential for immunogenic reactions. In addition to inhibiting ligand-dependent receptor signaling, cetuximab can also induce antitumor activity through antibody-dependent cellular cytotoxicity (ADCC) mediated by immune effector cells. Multiple prospective and retrospective studies have demonstrated that the addition of anti-EGFR monoclonal antibodies to standard doublet chemotherapy regimens (FOLFOX or FOLFIRI) improves response rates and survival outcomes in patients with *RAS*/*BRAF*wt metastatic CRC. Maintenance strategies following induction therapy are frequently implemented to reduce cumulative toxicity while maintaining disease control. Primary tumor location has also emerged as a relevant predictive factor, with left-sided tumors deriving the greatest clinical benefit from anti-EGFR-based combinations. In contrast, right-sided tumors appear to show less benefit from EGFR blockade and are often preferentially treated with anti-angiogenic-based strategies. Collectively, these findings have established anti-EGFR therapy combined with chemotherapy as a standard first-line option for patients with *RAS*/*BRAF*wt left-sided metastatic CRC [12].

The identification of predictive biomarkers for EGFR inhibitors in mCRC exemplifies the evolution of precision oncology in CRC. Initial patient selection strategies relied on EGFR expression assessed by immunohistochemistry. However, subsequent clinical trials demonstrated that EGFR expression alone did not reliably predict response to EGFR-targeted therapies. Succeeding molecular analyses revealed that activating mutations in *KRAS*, and later in *NRAS*, were strongly associated with primary resistance to anti-EGFR monoclonal antibodies. These findings led to the implementation of extended *RAS* testing as a mandatory prerequisite for treatment selection. Consequently, anti-EGFR therapies are currently restricted to patients with *RAS*/*BRAF*wt mCRC. Constitutive activation of RAS pathway, most commonly due to *KRAS* mutations, present in approximately 40–45% of CRCs, and less frequently *NRAS* mutations (5–7%), results in persistent downstream signaling independently of upstream EGFR activation. This constitutive signaling renders EGFR blockade ineffective, resulting in primary resistance to anti-EGFR monoclonal antibodies. *RAS* alterations may occur in *KRAS* exons 2, 3, and 4 and in NRAS exons 2 and 3; however, approximately 85% of cases involve mutations in *KRAS* exon 2, most commonly affecting codons 12 and 13. Similarly, *BRAF*V600E mutations, identified in 7–15% of CRC cases, drive MAPK pathway activation downstream of *RAS* and are associated with poor prognosis and limited benefit from anti-EGFR monotherapy, highlighting the importance of comprehensive molecular profiling before treatment selection [8,13].

During anti-EGFR therapy, acquired *RAS* mutations have been detected in up to 50–60% of patients who were initially classified as *RAS*wt. This phenomenon represents one of the most common mechanisms of secondary resistance and reflects clonal selection under therapeutic pressure. In addition, right-sided primary tumors consistently derive less clinical benefit from anti-EGFR-based strategies. This reduced efficacy is thought to reflect underlying biological differences, including a higher prevalence of intrinsic resistance mechanisms and enrichment of hyperselection-positive alterations. However, recent evidence, including the PARADIGM trial, has demonstrated that in molecularly hyperselected *RAS*wt populations identified through ctDNA profiling, anti-EGFR therapy can significantly improve overall survival compared with bevacizumab-based strategies, irrespective of tumor sidedness [18]. Increasing evidence indicates that selection based solely on *KRAS* and *NRAS* mutational status is insufficient to identify tumors truly dependent on EGFR signaling. Consequently, the concept of negative hyperselection has emerged involving the exclusion of additional genomic alterations to optimize patient selection. This approach expands molecular screening beyond *RAS* and *BRAF* testing to exclude additional genomic alterations associated with resistance, including *HER2* or *MET* amplification, *PIK3CA* exon 20 mutations, *MAP2K1* alterations, *PTEN* loss, and oncogenic kinase fusions. Clinical studies, such as PRESSING trial and PRESSING2 trial, have demonstrated that patients lacking these alterations derive significantly greater benefit from anti-EGFR therapy. These findings emphasize the importance of defining appropriate molecular panels and clinically meaningful allele-frequency thresholds in order to avoid the inclusion of tumors harboring subclonal resistance alterations [19,20].

The concept of “negative hyperselection” involves excluding additional alterations such as HER2 amplification, MET amplification, and PIK3CA mutations.

Nevertheless, several aspects of molecular hyperselection remain unresolved. In particular, the optimal composition of hyperselection panels, the definition of clinically meaningful mutant allele fraction thresholds for MAPK pathway alterations, and the predictive relevance of additional genomic events, such as *PIK3CA* mutations or *FGFR1* aberrations, have not yet been fully established [21,22]. Prospective clinical studies are required to establish their clinical relevance and guide more precise patient selection. Table 1 summarizes the main hyperselection panels published to date and the principal hyperselection strategies.

Growing evidence suggests that the overexpression of EGFR ligands, such as amphiregulin (AREG) and epiregulin (EREG), could be associated with enhanced sensitivity to anti-EGFR monoclonal antibodies rather than reduced responsiveness. This phenomenon appears to be driven by therapy-induced upregulation and competitive binding for the EGFR receptor [8,16,27]. Ongoing studies, including the ARIEL clinical trial, are investigating whether right-sided *RAS*wt mCRC with high AREG and EREG expression may represent a subgroup that still benefits from anti-EGFR therapy despite unfavorable primary tumor location [28,29].

One of the most frequently described mechanisms of acquired resistance involves alterations in the extracellular domain (ECD) of EGFR, which impair antibody binding without affecting ligand interaction or receptor signaling. Somatic mutations including *S492R*, *G465R*, *G465E*, and *S468R* have been identified at the antibody-binding interface and can confer resistance to cetuximab and, in some cases, panitumumab. Notably, *S492R* has been predominantly described following cetuximab exposure and is rarely observed after treatment with panitumumab. In addition, *R198*/*R200* methylation and the *EGFR* kinase domain mutation *V843I* have been associated with disease progression during cetuximab treatment. These mutations alter the epitope recognized by therapeutic antibodies, thereby preventing effective receptor blockade while preserving downstream signaling activity [16,30]. Molecularly guided therapeutic strategies are being investigated to overcome these resistance mechanisms, including agents targeting distinct epitopes within the EGFR extracellular domain to bypass acquired mutations. Examples of such approaches include the oligoclonal antibody MM-151 and the monoclonal antibody necitumumab. Preclinical studies have demonstrated the antitumor activity of MM-151, an oligoclonal antibody designed to bind multiple regions within the EGFR extracellular domain [31,32]. Necitumumab may retain activity against the *EGFR S468R* variant, providing a rationale for genotype-guided repurposing of this agent [33,34].

Although less frequent, *MET* amplification represents a well-established bypass mechanism of resistance that may emerge under therapeutic pressure during EGFR blockade. Several studies have reported that *MET* amplification can be detected in ctDNA prior to clinical relapse, accounting for approximately 1% of cases of acquired resistance to EGFR inhibitors. The receptor tyrosine kinase c-MET, encoded by the proto-oncogene *MET*, activates downstream signaling pathways including PI3K-AKT and RAS-RAF-ERK following binding to hepatocyte growth factor (HGF), thereby promoting cellular proliferation and survival [16,35]. Accordingly, c-MET inhibitors are currently being investigated as a strategy to overcome this resistance mechanism. The ongoing OrigAMI-2 trial is a phase III trial evaluating the combination of chemotherapy and amivantamab, a bispecific antibody targeting MET and EFGR, as a first-line treatment for *BRAF*/*RAS*wt mCRC [36].

Another emerging strategy involves the development of bispecific antibodies targeting multiple oncogenic pathways. Increasing evidence supports the presence of a subpopulation of stem-like tumor cells expressing the receptor 5 coupled to the leucine-rich G-repeat protein (LGR5), which exhibit self-renewal capacity, drive tumor growth and metastasis, and contribute to therapeutic resistance. Petosemtamab is a novel bispecific antibody targeting both LGR5 and EGFR currently under clinical investigation. Its mechanism of action involves simultaneous inhibition of EGFR signaling and targeting of LGR5-positive tumor-initiating cells, which are implicated in tumor propagation and therapeutic resistance. In addition to blocking ligand-dependent signaling, petosemtamab enhances EGFR receptor internalization and degradation in LGR5+ cells, and may promote Fc-mediated activation of the innate immune system through antibody-dependent cellular phagocytosis (ADCP) and enhanced ADCC. This dual targeting strategy aims to overcome both signaling-driven resistance and tumor cellular heterogeneity. While preliminary data is promising, further validation in advanced-phase trials is required [37,38].

Malignant tumors are characterized by both spatial and temporal heterogeneity, a phenomenon particularly relevant in mCRC. Tissue biopsies used for genomic analysis may be limited by sampling bias due to their invasive nature and the difficulty of capturing multiple metastatic sites. Compared to traditional tissue biopsies, ctDNA analysis provides a more comprehensive and dynamic representation of all tumor clones, offering broader insights than a single biopsy. mCRC clonal populations can vary due to tumor microenvironment and treatment pressures. The use of ctDNA allows us to track clonal evolution secondary to the treatments in real-time to improve precision medicine [12]. Importantly, ctDNA analysis is increasingly transitioning from a research tool to a clinically actionable biomarker that enables real-time treatment adaptation and patient selection in mCRC [39].

ctDNA analysis has arisen as a critical tool to guide anti-EGFR rechallenge strategies. The rechallenge is as a potential therapeutic option for patients with chemorefractory *RAS*wt mCRC after a period of anti-EGFR-free therapy [40]. It has been evaluated for implementation as a third-line treatment onwards for those patients who achieved a significant initial clinical response to anti-EGFR inhibitors. The biological rationale is based on intratumorally heterogeneity and clonal evolution. During a treatment-free interval, the selective pressure is removed, potentially leading to a regression of resistant clones. This shift in clonal architecture may restore the tumor’s sensitivity to the EGFR inhibitor. To optimize patient selection, longitudinal ctDNA monitoring enables real-time assessment of these dynamic clonal changes, allowing identification of patients in whom resistant clones have decayed below detectable thresholds, thereby quantifying the molecular landscape prior to re-exposure [39,41,42]. The decay of the mutant allele frequency (MAF) of *RAS*, and other resistant clones, in ctDNA during non-EGFR-based treatment has been estimated to have a half-life ranging between 3.7 and 4.7 months. These findings suggest clonal evolution during therapy [43]. This time has been used in the past to empirically test EGFR inhibitors rechallenge, with low objective response rates (ORRs). ctDNA has demonstrated significant suitability for identifying patients eligible for rechallenge [44,45]. This concept was initially proven in 2015, demonstrating that individuals benefiting from multiple anti-EGFR treatments exhibited fluctuating levels of ctDNA *RAS* mutations. Subsequent trials, including CRICKET trial, retrospectively confirmed that having *RAS*wt ctDNA at the time of rechallenge was a mandatory condition for a positive response. The multi-center phase II CRICKET evaluated a rechallenge strategy using cetuximab and irinotecan in patients with *RAS* and *BRAF*wt mCRC who had acquired resistance to first-line irinotecan- and cetuximab-based therapy [46]. Among the 28 patients who were enrolled, there was an ORR of 21%, with six patients achieving partial responses and nine experiencing disease stabilization. A retrospective analysis of baseline ctDNA revealed a correlation between the presence of *RAS* mutations and shorter progression-free-survival (PFS), underscoring the necessity of using ctDNA in selecting patients for this rechallenge approach. Subsequent prospective studies, notably the CHRONOS phase II, involved screening patients with tissue *RAS*wt tumors previously treated with anti-EGFR therapy through ctDNA [47]. ctDNA-based screening led to the exclusion of approximately 31% of patients due to the detection of resistance-associated mutations. Among the patients included in the study, 63% achieved disease control, suggesting that ctDNA-guided anti-EGFR rechallenge may represent a feasible and potentially effective strategy for patients with refractory mCRC. Available evidence suggests that low-frequency resistance mutations may not significantly compromise responses to anti-EGFR rechallenge, although the optimal allele frequency threshold remains uncertain. In a retrospective analysis, the relative frequencies of mutant alleles (rMAF) were defined as the ratio between the MAF of the resistance mutation and the highest detected MAF detected in the sample. Patients with rMAF ≤12.4% demonstrated significantly longer PFS (6.1 vs. 2.6 months; *p* = 0.04), and a numerically higher OS (28.3 vs. 3.7 months; *p* = 0.09) [48,49,50].

To overcome clonal escape and restore tumor dependency on EGFR signaling, there is current investigation to integrate ctDNA-guided molecular profiling with combination therapies targeting both EGFR and resistance-associated pathways [51]. For instance, the ongoing OrigAMI-1 trial (NCT05379595) is evaluating an enhanced rechallenge strategy using the bispecific EGFR/MET antibody amivantamab in patients with anti-EGFR–pretreated mCRC. The study incorporates ctDNA-based screening to identify resistance alterations and guide therapeutic targeting of bypass signaling pathways.

## 3. Drug Discovery in BRAFV600 mCRC Patients

*BRAF* mutations occur in approximately 8–12% of mCRC patients [52]. The *V600E* mutation represents 95% of all *BRAF* mutations, and it is associated with female sex, nodal and peritoneal spread, right-sided tumors and mucinous tumors with poor differentiation. About 30% of the *BRAF*-mutated patients also present MSI, and they are usually mutually exclusive with *KRAS* mutations. *BRAF* mutations can be classified in three categories based on their function on *BRAF* dimerization. Class I mutations have activity as monomers and contain *V600E*, *V660K*, *V600D*, *V600M*, and *V600R*. Class II mutations are constitutively active only as dimers and include *L597Q*/*R*/*S*/*V*, *G464V*/*E*, *G496A*/*V*/*R*, *K601 E*/*NT*, and *P367 L*/*S.* Class III mutations require coexisting *RAS* activation because they have altered kinase activity, while class I and II mutations are both *RAS*-independent and activate the MAPK pathway. Class III mutations contain *D594G*, *D594N*, *G466E*, and *G466V*. The non-*V600E* mutations present a similar prognosis as *RAS*/*BRAF*wt mCRC, and some evidence suggests they might benefit from anti-EGFR therapy [53].

The prognosis for *BRAF*-mutated mCRC has significantly improved due to the discovery of targeted therapy. Before the targeted therapy era, treatment recommendations for patients with *BRAF*-mutant disease were based on outcomes in subgroups of several clinical trials with a median overall survival (OS) of 11 months, and poor response to standard chemotherapy. The main BRAF inhibitors are encorafenib, vemurafenib, and dabrafenib [12]. First, BRAF blockade in monotherapy was evaluated in melanoma patients, with vemurafenib, evidencing response rates of 48% vs. 5% in the dacarbazine arm [54]. Subsequently, the VE BASKET trial (NCT01524978) evaluated vemurafenib efficacy in nonmelanoma cancers, observing activity in non-small cell lung cancer (NSCLC) with a response rate of 42% [55]. However, a phase I trial had evidenced that vemurafenib alone had insufficient activity in patients with *BRAF V600E* mCRC [56]. Emerging laboratory data suggested that it might be mediated through feedback activation of EGFR signaling, driven by the loss of ERK-dependent negative feedback [57]. It was evidenced that combination of RAF and EGFR inhibition blocked reactivation of MAPK signaling in *BRAF* mutant CRC cells, improving efficacy in vitro and in vivo [58]. According to these findings, the CRC cohort in the VE BASKET trial was amended to include vemurafenib plus and EGFR inhibitor (cetuximab), but the results were discouraging (overall response rate 4%). Since then, several trials were conducted combining BRAF inhibitors with EGFR inhibitors, MEK inhibitors (such as trametinib, binimetinib and cobimetinib) or chemotherapy, with modest results [8,52].

The phase III BEACON trial was the largest trial that confirmed the combination of encorafenib plus cetuximab as the standard of care for *BRAF-V600E* mCRC after at least one previous line of systemic therapy [59]. It evaluated the combination of encorafenib/cetuximab with or without binimetinib vs. irinotecan/cetuximab-based chemotherapy. Updated post hoc analysis confirmed that both the triplet and doublet arms demonstrated better ORR (26.8% for the triplet, 19.5% for the doublet and 1.8% for control), PFS (4.3 months for the triplet, 4.2 months for the doublet, and 1.5 months for control), and OS than the chemotherapy arm (9.3 months for both the triplet and the doublet and 5.9 months for control). It has been reported that tumors with stronger immune signature (for example, increased T cells) showed a trend towards increased benefit from treatment with the triplet with binimetinib [60]. Subsequently, the phase III BREAKWATER trial has evaluated the use of cetuximab and encorafenib administered either with or without chemotherapy (mFOLFOX6 or FOLFIRI) as first-line treatment. Compared to standard care, the combination of encorafenib, cetuximab and mFOLFOX6 significantly increased PFS (median, 12.8 vs. 7.1 months; hazard ratio (HR) for progression or death, 0.53; *p* < 0.001). The OS was significantly improved compared to standard of care according to the latest reported data (30.3 vs. 15.1 months; HR for death, 0.49; *p* < 0.001) [61]. These findings have constituted a major shift in the paradigm of first-line treatment of *BRAF*-mutated mCRC.

On the other hand, biomarker-guided research is ongoing to select those patients that could be associated with a better response to anti-BRAF/EGFR therapy. It has been proven that inactivating mutations in *RNF43*, a negative regulator of WNT, predicts improved response rates and survival outcomes in patients with microsatellite-stable (MSS) tumors. In patients with *MSS-RNF43*-mutated versus *MSS-RNF43*-wt tumors, a statistically significant benefit was observed in both progression-free survival (HR, 0.30; 95% confidence interval (CI), 0.12–0.75; *p* = 0.01) and overall survival (HR, 0.26; 95% CI, 0.10–0.71; *p* = 0.008) [62]. Moreover, it has been evaluated that plasmatic *BRAF* allele fraction (AF) has a prognostic role for survival in mCRC treated with BRAF inhibitors, acting as a surrogate of tumor burden and aggressiveness. An exploratory analysis of predictive value showed that high-*BRAF* AF patients could benefit more from treatment intensification with triplet therapy (BRAF, EGFR and MEK inhibitors) than low-BRAF AF patients [63]. Consistently, comprehensive molecular profiling from the BEACON trial highlighted the complex genomic landscape reporting that acquired resistance commonly converges on reactivation of the MAPK pathway through alterations such as *KRAS*, *NRAS*, *MAP2K1* mutations or *MET* amplifications [60].

There are several trials ongoing about rechallenge with BRAF inhibitors, such as the phase II REFISH trial (NCT07178717) [64]. The aim is to evaluate the efficacy of encorafenib plus cetuximab as a rechallenge strategy. It has the same background as the anti-EGFR rechallenge: reintroduce BRAF inhibitors among patients who have previously responded to such strategy after a free-interval period. The combination of clinical features such as long-lasting response, and extended time off therapy, in combination with the absence of molecular mechanism of resistance in ctDNA, and new biomarkers such as *RNF43* mutation or the plasmatic *BRAF* AF, may help identify those patients who may achieve the greatest benefit from rechallenge [65,66].

Recent clinical trials combining immunotherapy with BRAF inhibitors in *BRAFV600E* mCRC have demonstrated encouraging outcomes. In MSS *BRAFV600E* mCRC, a phase I/II trial (NCT04017650) using encorafenib, cetuximab, and nivolumab had promising results [67]. In a similar direction, the combination of trametinib, dabrafenib, and spartalizumab (anti-PD1) showed durable responses (NCT03668431) [68]. For patients, with *BRAFV600E*-*MSI* mCRC, there is an ongoing randomized phase II trial (NCT05217446) comparing the efficacy of the combination of pembrolizumab with encorafenib and cetuximab versus pembrolizumab alone in first line [69]. Mechanistically, BRAF/MEK inhibition works in concert with immune checkpoint blocking by inducing antigen-presenting mechanisms and activating CD8+ T cells in the tumor microenvironment [70]. While complement system and CD8+ T-cell activation were linked to resistance, responders showed increased immunological activation and MAPK signaling signatures [71]. These results imply that patient selection for these combination therapies may be optimized by baseline immunological profile.

The main resistance mechanisms described after BRAF inhibitors combination therapy include *MAP2K1*, *GNAS*, *ARAF*, *PTEN*, *ERBB2*, *MEK*, and *EGFR* mutations, as well as *KRAS*, *MET*, and *EGFR* amplifications [52,72]. The analysis from ctDNA in the BREAKWATER trial revealed that for acquired alterations in *KRAS*, *NRAS*, and *MAP2K1* mutations, *MET* amplifications and *BRAF* exon deletions were less frequent in the encorafenib + cetuximab + mFOLFOX6 group than in the group without chemotherapy. These findings support the greater therapeutic effects of targeted therapy and cytotoxic chemotherapy in avoiding resistance mechanisms [73]. Current studies are evaluating several combinations to overcome resistance. Azanosertib (ZN-c3) is a novel, selective, orally bioavailable WEE1 inhibitor that enhances tumor growth inhibition with BRAF/EGFR inhibitors in *BRAF V600E* CRC xenograft models. The ongoing phase I/II trial (NCT05743036) is exploring the combination of encorafenib + cetuximab + azanosertib in *BRAF*-mCRC previously treated with one or two treatment regimens [74]. Another strategy is the new pan-RAF inhibitors in combination with MEK inhibitors to overcome resistance [75,76,77]. For example, PF-07799933 is a next-generation, selective, brain-penetrant, pan-mutant BRAF inhibitor that has been combined with MEK inhibitors in a phase I trial [78]. In addition, BRAF degraders are based in avoiding paradoxical RAF activation, and the degraded protein can no longer incorporate into a dimeric signaling complex [79,80]. For example, CFT1946 is an oral drug that specifically binds BRAFV600X, inducing the ubiquitination and degradation via E3 ubiquitin ligase complex. A phase I/II trial is ongoing to evaluate CFT1946 as monotherapy and in combination with trametinib or cetuximab in subjects with *BRAF V600* mutant solid tumors [81]. There are novel *BRAFV600E* inhibitors that are designed to evade the paradoxical MAPK activation, named “paradox breakers” [82]. There are several in current evaluation such as plixorafenib [83] or RG6344 [84]. Finally, preclinical studies suggest that the overexpression and activation of vascular endothelial growth factor A (VEGFA) may contribute to resistance to BRAF inhibitors in CRC. The ongoing BRAVE trial (NCT06411600) is a phase II trial to evaluate the efficacy of adding bevacizumab (VEGA inhibitor) to the combination of encorafenib plus cetuximab in *BRAF-V600E* mCRCs that have progress after one or two prior chemotherapy regimens [85].

**Figure 2:**
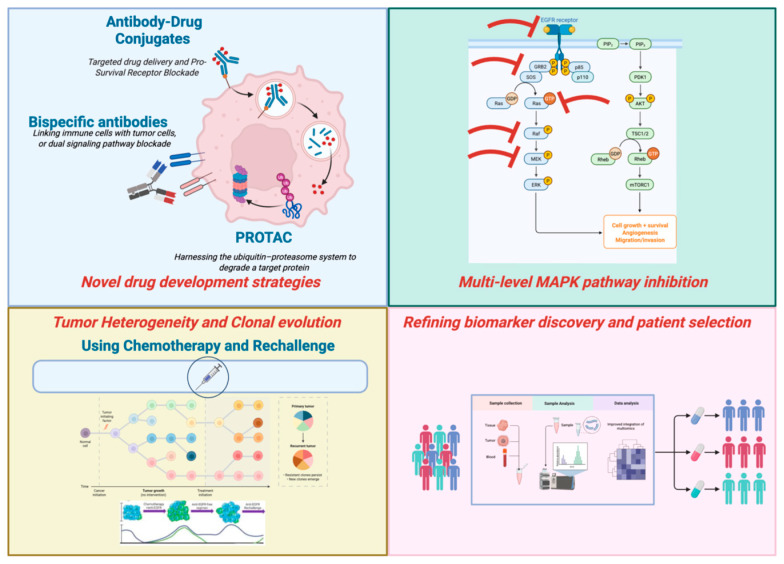
Conceptual framework of emerging strategies to overcome resistance in molecularly selected metastatic colorectal cancer. The figure illustrates key therapeutic approaches currently being developed to counteract intrinsic and acquired resistance mechanisms that have been commented in the review. These include antibody–drug conjugates (ADCs), bispecific antibodies targeting multiple receptors, and PROTAC-based degradation strategies; vertical inhibition of the MAPK pathway at multiple nodes; integration of chemotherapy and rechallenge strategies to suppress clonal evolution; and refinement of biomarker-driven patient selection to optimize treatment sequencing and personalize combination therapies.

As illustrated in Figure 2, the development of next-generation strategies, including vertical blockade and novel drug constructs, aims to preemptively address these convergent resistance pathways across different molecular subsets.

## 4. KRAS Inhibition in mCRC: From KRASG12C to Next-Generation Strategies

*KRAS*, together with *HRAS* and *NRAS*, belongs to the *RAS* family of small GTPases that cycle between an inactive GDP-bound state and an active GTP-bound conformation, a process regulated by GTPase-activating proteins (GAPs), such as NF1, and guanine nucleotide exchange factors (GEFs), including SOS1 [86,87]. In its active GTP-bound state, *KRAS* interacts with downstream effectors such as *BRAF* and *CRAF*, initiating signaling through the MAPK cascade. Oncogenic *KRAS* mutations impair intrinsic GTP hydrolysis, thereby stabilizing the active GTP-bound conformation and resulting in constitutive pathway activation.

*KRAS* is one of the most frequently mutated oncogenes in human cancer, occurring in approximately 20% of all malignancies [88]. In mCRC, *KRAS* mutations are detected in approximately 40–50% of the patients. The majority of pathogenic alterations occur at codons 12 or 13, most commonly resulting in *G12D*, *G12V*, and *G12C* mutations. *KRAS* was historically considered an undruggable target until the identification of a cryptic binding pocket adjacent to cysteine 12 in *KRAS G12C*, which enabled the development of covalent inhibitors that selectively lock the protein in its inactive GDP-bound state [89]. *KRAS G12C* mutations occur in approximately 3–4% of patients with mCRC. It has been associated with poorer outcomes compared with other *KRAS*-mutant subtypes when treated with standard chemotherapy. In fact, OS among patients with *KRAS G12C* tumors was 16.1 months in first-line treatment, compared to 18.3 months in patients with non-*G12C KRAS*-mutated tumors [90,91,92,93]. KRAS G12C inhibitors, including sotorasib and adagrasib, were the first agents of this class to enter clinical development and showed remarkable efficacy as monotherapy in NSCLC. However, their activity in mCRC has been more limited, with an ORR of approximately 9% for sotorasib and 19% for adagrasib [94,95,96,97,98]. This reduced efficacy is largely explained by adaptive feedback activation of EGFR signaling, a resistance mechanism analogous to that observed in *BRAF*-mutant CRC, where EGFR-mediated pathway reactivation compromises targeted inhibition. In *KRAS G12C*-mutant mCRC, *EGFR* activation promotes signaling through *RAS*wt isoforms, thereby restoring downstream MAPK signaling and bypassing *KRAS* blockade [99]. Preclinical studies demonstrated that simultaneous inhibition of *KRAS G12C* and *EGFR* enhances antitumor activity, and clinical trials combining KRAS G12C inhibitors with anti-EGFR monoclonal antibodies such as cetuximab or panitumumab have reported response rates two- to three-fold higher than those observed with monotherapy [100,101,102,103,104]. Consistent with these findings, the phase III CodeBreaK 300 trial evidenced that the combination of sotorasib and panitumumab improved PFS and ORR compared with standard later-line treatments (regorafenib or TAS-102) in patients with refractory *KRAS G12C*-mutant mCRC, although overall survival data remain immature [105].

KRASG12C inhibitors are specifically designed to inhibit this allele but are not active against other prevalent *KRAS* mutations such as *G12D* or *G12V* due to covalent binding to the cysteine residue [32]. Multiple mechanisms of acquired resistance to KRAS G12C inhibitors have already been described, including secondary *KRAS* alterations such as *G12D*/*R*/*V*/*W*, *G13D*, *Q61H*, *R68S*, *H95D*/*Q*/*R*, *Y96C*, and high-level amplification of the *KRASG12C* allele. Acquired bypass mechanisms of resistance include *MET* amplification; activating mutations in *NRAS*, *BRAF*, *MAP2K1*, and *RET*; oncogenic fusions involving *ALK*, *RET*, *BRAF*, *RAF1*, and *FGFR3*; and loss of-function mutations in *NF1* and *PTEN* [106]. Biomarker insights from paired plasma samples in the phase III CodeBreaK-300 trial identified countless mechanisms of resistance, such as alterations in *TP53*, *DNMT3A*, *ERBB2*, and *LRP1B*, and increases in *KRAS* copy number variations [107]. This suggests that resistance arises through multiple complex mechanisms, underscoring the complexity of resistance in *KRASG12C*-mutant mCRC.

Combining therapies has emerged as a key strategy to overcome primary and acquired resistance in *KRAS* mutated mCRC [9]. In particular, the combination of KRAS inhibitors with anti-EGFR monoclonal antibodies and other targeted agents, such as SHP2, SOS1 or MEK inhibitors, are being explored (e.g., KRYSTAL-2 and 14, CodeBreaK 101, NCT05288205, NCT06024174, NCT05578092) [52]. Downstream, CDK4/6 co-targeting (e.g., KRYSTAL-16 with palbociclib) leverages the KRAS–Cyclin-D axis, with promising preclinical data [108,109]. This approach is based on the idea of vertical pathway blockade; by concurrently focusing on several proteins in the MAPK signaling cascade, it would reduce the compensatory signaling and retard the development of resistance mechanisms [110]. Other strategies, such as the combination with cytotoxic chemotherapy, are being also explored. For example, the phase III CodeBreak 301 trial is investigating first-line therapy with FOLFIRI combined with the KRASG12C inhibitor sotorasib and panitumumab, while the KANDLELIT-012 trial is evaluating FOLFOX in combination with the KRASG12C inhibitor, MK-1084, and cetuximab. These approaches aim to achieve more comprehensive suppression of oncogenic signaling and delay resistance emergence by simultaneously targeting multiple levels of the signaling network [111,112].

Beyond *KRAS G12C*, emerging RAS-targeted therapies aim to address a wider spectrum of *KRAS* alterations. *KRAS G12D* represents the most common *KRAS* mutation, accounting for approximately 25–45% in mCRC [113,114,115]. Novel agents targeting this variant, such as MRTX1133, a selective, non-covalent small-molecule, have demonstrated strong antitumor activity in preclinical models. Early-phase clinical trials evaluating these agents in humans are currently underway [116,117,118]. Approaches to overcome resistance mechanisms include mutation-specific inhibitors targeting non-*G12C KRAS* variants, but also pan-KRAS inhibitors designed to suppress multiple *KRAS* mutant forms, and broader pan-RAS inhibitors [119]. Pan-RAS inhibitors are currently under clinical development. These agents aim to inhibit multiple *RAS* isoforms, including mutant *KRAS*, *NRAS*, and *HRAS*, and in some cases may also target wild-type *RAS* signaling. RMC-6236 (daraxonrasib) represents a demonstrative compound within this emerging class. It binds a conserved switch-pocket across RAS isoforms. Early-phase clinical updates reported promising tolerability and initial tumor responses in NSCLC and pancreatic cancer, including an ORR of ~20% in pancreatic cancer [120,121,122]. Broad KRAS-selective inhibitors have been developed to target multiple mutant *KRAS* isoforms while sparing *KRAS*wt and other RAS family members, thereby potentially minimizing off-target toxicity. BI 2865, a novel non-covalent inhibitor that selectively binds the inactive conformation of *KRAS*, has demonstrated broad preclinical activity across a range of *KRAS* mutations, supporting the feasibility of pan-KRAS inhibition as a therapeutic strategy in *KRAS*-driven malignancies [123]. Although early clinical data are preliminary, these approaches hold promise for overcoming both intrinsic and acquired resistance in *RAS*-driven mCRC [110,124].

## 5. HER2 Inhibitors in HER2 Positive mCRC Patients

HER2, encoded by the *ERBB2* gene, is a member of the ERBB family of receptor tyrosine kinases. It plays a central role in regulating cell survival, proliferation, and differentiation. Unlike other ERBB receptors, HER2 lacks a known ligand and primarily functions through heterodimerization with other ERBB family members. This mechanism result in potent activation of downstream signaling pathways, including the MAPK and PI3K-AKT cascades [8].

*ERBB2* amplification, which typically results in HER2 protein overexpression, is detected in approximately 2–5% of patients with mCRC. It is observed predominantly in *RAS*/*BRAF*wt tumors and more frequently in distal locations such as rectal cancers. This alteration has been proposed, based on retrospective data, as a negative predictive biomarker of response to anti-EGFR monoclonal antibodies [125,126]. However, *HER2* amplification also defines a distinct molecular subgroup that may benefit from HER2-targeted therapeutic strategies [127].

*HER2* overexpression or gene amplification is typically assessed using a combination of immunohistochemistry (IHC) and fluorescence in situ hybridization (FISH). According to established criteria, tumors are considered HER2-positive when they demonstrate an IHC score of 3+, defined as strong and complete membranous staining in ≥10% of tumor cells. In cases with an equivocal IHC score of 2+, confirmatory FISH analysis to assess *ERBB2* gene amplification is required [128].

Several HER2-targeted therapies have demonstrated clinical activity in HER2-positive mCRC, including monoclonal antibodies (trastuzumab, pertuzumab), tyrosine kinase inhibitors (lapatinib, tucatinib, neratinib), and antibody–drug conjugates (trastuzumab-deruxtecan) [129,130]. Dual HER2 blockade has emerged as an effective therapeutic strategy in this setting. The clinical relevance of HER2 as a therapeutic target in mCRC was first demonstrated in the phase II HERACLES-A trial, a proof-of-concept study evaluating dual HER2 blockade with trastuzumab and lapatinib in heavily pretreated HER2-positive, KRASwt mCRC patients. This study showed meaningful clinical activity, achieving an ORR of 28%. It established HER2 amplification as a valid actionable target in mCRC and paved the way for subsequent HER2-directed strategies. Similarly, the phase IIa MyPathway trial reported an ORR of 32% with trastuzumab plus pertuzumab, while the phase II MOUNTAINEER study observed an ORR of 38.1% with trastuzumab and tucatinib in the same population [131]. Antibody–drug conjugates have further expanded therapeutic options in this molecular subgroup. The phase II HERACLES-B trial evaluated trastuzumab emtansine (T-DM1) in combination with pertuzumab in patients with *RAS*/*BRAF*wt, HER2-positive refractory mCRC. Although the primary endpoint was not met, PFS was comparable to previous HER2-targeted therapy studies. Notably, the phase II DESTINY-CRC01 trial demonstrated activity of trastuzumab-deruxtecan in patients with HER2-positive mCRC refractory to standard therapies, achieving an ORR of 45.3%. Responses were observed regardless of prior anti-HER2 exposure, while no activity was detected in HER2-low tumors, confirming the predictive value of HER2 amplification for response to this agent [132].

Overall, the most frequently described resistance mechanisms include activation of bypass signaling pathways involving *RAS*/*RAF*, *EGFR*, and *MET* alterations, activating mutations in the PI3K/AKT pathway, and alterations in genes such as *CDK12* and *NOTCH*. In patients treated with trastuzumab and tucatinib, acquired *HER2* mutations affecting the kinase domain and extracellular regions, including *L755S*, *V777L*, *D769Y*, *G727A*, and *S310F*, have also been reported. It highlights the dynamic molecular evolution under HER2-targeted therapy [133].

Emerging evidence suggests that resistance mechanisms to HER2-targeted therapies in mCRC vary depending on the therapeutic modality. In the DESTINY-CRC01 study, trastuzumab-deruxtecan retained antitumor activity even in a subset of tumors harboring activating *RAS* or *PIK3CA* mutations, many of which were likely acquired following prior anti-EGFR therapy. Although limited by sample size, these findings contrast with earlier studies of dual HER2 blockade, such as HERACLES-A and MyPathway, where baseline *KRAS* or *PIK3CA* mutations were associated with significantly reduced response rates, supporting the role of MAPK and PI3K pathway reactivation as key resistance mechanisms. The preserved activity of trastuzumab-deruxtecan may be explained by its distinct mechanism of action, which combines HER2 signaling inhibition with intracellular delivery of a cytotoxic payload [131].

To overcome potential acquired resistance mechanisms, combination strategies incorporating chemotherapy are also being investigated in HER2-positive mCRC. Preliminary first-line results with the dual HER2-targeting bispecific antibody zanidatamab (NCT06695845), which inhibits HER2 dimerization, in combination with mFOLFOX6 ± bevacizumab, demonstrated a confirmed ORR of 91% (10 of 11 evaluable patients achieving partial responses), with a manageable safety profile [134]. In addition, the ongoing phase III MOUNTAINEER-03 trial is evaluating the combination of tucatinib, trastuzumab, and mFOLFOX6 as first-line therapy for patients with HER2-positive mCRC [135]. The rapid expansion of the clinical trial landscape reflects the shift towards more personalized and biomarker-driven regimens.

The most relevant published and ongoing trials that are currently redefining the standard of care for molecularly selected mCRC are summarized in Table 2. Given the clinical relevance of the resistance mechanisms, Table 3 provides an integrated overview of molecular alterations.

## 6. Discussion and Conclusions

The therapeutic landscape of mCRC has evolved from empiric cytotoxic sequencing toward a biologically stratified framework driven by actionable genomic alterations and biomarker selection of patients. Molecularly defined subgroups, including *RAS*/*BRAF*wt, *BRAFV600E*-mutant, *KRAS*-mutant, and *HER2*-amplified tumors, have demonstrated that oncogene-directed strategies can meaningfully improve clinical outcomes when rational drug combinations are employed. However, across molecular subsets, the durability of clinical benefit remains limited by intrinsic heterogeneity and the emergence of adaptive mechanisms of resistance. A recurrent observation across targeted therapy development in mCRC is the convergence of resistance mechanisms on common signaling pathways. Under selective pressure from *EGFR*, *BRAF*, *KRAS*, or *HER2* inhibition, tumors frequently restore MAPK or PI3K-AKT signaling through multiple mechanisms, including downstream pathway reactivation, receptor-level alterations, bypass signaling mediated by parallel receptor tyrosine kinases such as *MET* or *HER2*, and the expansion of pre-existing resistant subclones. These patterns indicate that resistance to target therapy is not a purely stochastic process but rather a biologically constrained evolutionary phenomenon driven by selective pressure under treatment [138]. Despite extensive genomic heterogeneity, diverse resistance alterations frequently converge on a limited number of critical signaling pathways, predominantly the MAPK and PI3K-AKT axes. Traditionally, therapeutic approaches have focused on suppressing dominant oncogenic drivers, such as EGFR or BRAF, often leading to transient responses followed by the emergence of resistance. However, this paradigm is increasingly being replaced by a more dynamic and predictive strategy aimed at anticipating mechanisms of adaptive escape. This includes the use of rational combination therapies such as vertical pathway inhibition, horizontal co-targeting of compensatory signaling nodes, emerging drug designs, targeted protein degraders, or antibody–drug conjugates. The integration of chemotherapy backbones with targeted agents is also emerging as a rational strategy in earlier treatment lines. This approach may limit polyclonal expansion and delay molecular escape, particularly in biologically aggressive subgroups such as *BRAFV600E*-mutant disease, where clinically meaningful benefit has been demonstrated.

To better characterize resistance phenotypes and guide personalized treatment strategies, future research should prioritize the integration of multi-omics data, including genomic, transcriptomic, and epigenetic information, to refine biomarker discovery and patient selection [139]. Resistance in CRC is not exclusively genomic; transcriptional reprogramming, tumor microenvironment dynamics, immune contexture, and metabolic adaptation all contribute to therapeutic failure. Comprehensive molecular characterization is therefore essential to capture both genetic and non-genetic determinants of treatment response. This paradigm shift also requires reconsidering how molecular testing is integrated into both clinical research and routine practice. Rather than sequentially evaluating individual biomarkers at predefined therapeutic decision points, comprehensive tumor profiling should be performed at diagnosis. The incorporation of ctDNA further transforms precision oncology into a dynamic process. Longitudinal ctDNA analyses enable real-time monitoring of clonal evolution, detection of emerging resistance alterations, and the implementation of molecularly guided rechallenge strategies. However, several barriers remain to its full clinical implementation, including cost, limited sensitivity in low-shedding tumors, and the lack of standardized Variant Frequency (VAF) thresholds to define clinically meaningful resistance mutations. Addressing these limitations will be critical to enabling the full clinical implementation of adaptive, biomarker-driven treatment algorithms.

Ultimately, the next frontier in mCRC management lies in integrating comprehensive molecular characterization with rational combination strategies and longitudinal surveillance. Such an approach moves the field beyond static molecular categorization toward continuous biological management of disease evolution. The future therapeutic landscape in mCRC will depend on the integration of biological strong observations to biomarker discovery, allowing the delivering of the right combination, at the right time, in the right patient.

## Figures and Tables

**Table 1:** Main studies using hyperselection panels.

Study	Population	Strategy of Hyperselection	mOS (months)	References
PRESSING [19]	1st line anti-EGFR (*n* = 94) *RAS*/*BRAF* WT	*HER2*/*MET* amp, *PIK3CA* exon 20 mut, *NTRK*/*ROS1*/*ALK*/*RET* fus, pMMR	17.3 vs. 15.2	Cremolini et al., Ann Oncol 2018
PRESSING [20]	Anti-EGFR any line (*n* = 650) RAS/BRAF WT, MSS, POLE ED WT, PRESSING negative	*NTRKmut*, *ERBB3*, *NF1mut*/*loss*, *MAP2K1*/*2mut*, *AKT2* mut, *PTEN*/*NF1* loss; *ERBB3*, *FGFR2*, *IGF1R*, *KRAS*, *ARAF*, *AKT1-2* amp; *EGFR* rearrangements	49.9 vs. 22.6	Randon et al., JCO Precis Oncol 2022
PANDA [23]	Phase II FOLFOX + Pan vs. 5FU + Pan (*n* = 147) *RAS*/*BRAF* WT, elderly population	PRESSING PANEL + *MAP2K1*, *PTEN* mut	29.5 vs. 20	Lonardi et al., Clin Colorectal Cancer 2023
PANAMA [24]	Phase III mFOLFOX6 + Pan → 5FU + Pan (*n* = 202) *RAS* WT, maintenance	*KRAS*, *NRAS*, *BRAF* (V600E), *AKT1*, *ERBB2*, *PIK3CA* exon 9/20, *PTEN*, *ALK* mut; *HER2*/*neu* amp	28.7 vs. 22.2	Stahler et al., Clin Cancer Res 2024
FIRE-3 [25]	Phase III FOLFIRI + Cet vs. FOLFIRI + Bev (*n* = 171) *RAS*/*BRAF* WT, pMMR	PRESSING1/PRESSING2 (≈7.6% alterations in PRESSING-neg)	38.5 vs. 27.5	Weiss et al., Eur J Cancer 2025
PARADIGM [26]	Phase III FOLFOX + Pan vs. FOLFOX + Bev (*n* = 733) *RAS* WT (basal tissue)	*PTEN*/*EGFR*/*KRAS*/*BRAF* mut, *HER2*/*MET* amp, *ALK*/*RET*/*NTRK* fus, MMR	41.4 vs. 18.7	Shitara et al., Nat Med 2024

Abbreviations: Amp, amplification; Mut, mutation; pMMR, proficient mismatch repair; Pan, Panitumumab; Cet, Cetuximab; Bev, Bevacizumab; Fus, Fusion.

**Table 2:** Clinical trials exploring combination of chemotherapy/immunotherapy with target therapy.

Clinical Trial	Population	Drugs	Phase	mOS (Months)	Location
OrigAMI-2 trial (NCT06662786) [36]	1st line *RAS*/*BRAF* WT, unresectable or left-sided mCRC.	Amivantamab + mFOLFOX6 or FOLFIRI vs. Cetuximab + mFOLFOX6 or FOLFIRI	Phase III	Ongoing	United States, Belgium, Brazil, Canada, China, France, Germany, Hungary, India, Israel, Italy, Japan, Malaysia, The Netherlands, Poland, Puerto Rico, South Korea, Spain, Sweden, Taiwan, Turkey, United Kingdom
BREAKWATER (NCT04607421) [61]	1st line *BRAF V600E* mCRC	Encorafenib + Cetuximab +/− mFOLFOX6 or FOLFIRI vs. SOC	Phase III	EC + mFOLFOX6: 30.3 vs. 15.1 EC + FOLFIRI (immature data): 10.5 vs. 10.3	United States, Argentina, Australia, Belgium, Brazil, Bulgaria, Canada, China, Czechia, Denmark, Finland, Germany, India, Italy, Japan, Mexico, The Netherlands, New Zealand, Norway, Poland, Russia, Slovakia, South Africa, South Korea, Spain, Sweden, Taiwan, Ukraine, United Kingdom
SWOG S1406 (NCT02164916) [136]	2nd or 3rd line *BRAF V600E* mCRC	Irinotecan + Cetuximab +/− Vemurafenib	Phase II	9.6 vs. 5.9	United States
SEAMARK (NCT05217446) [68]	1st line *BRAFV600E* and *MSI* mCRC	Pembrolizumab + Cetuximab + Encorafenib vs. Pembrolizumab	Phase II	Ongoing	United States, Australia, Belgium, Canada, Czechia, Denmark, France, Germany, Italy, The Netherlands, Norway, Poland, Slovakia, Spain, United Kingdom
CodeBreak 301 (NCT06252649) [111]	1st line *KRASG12C* mCRC	Sotorasib + Panitumumab + FOLFIRI vs. FOLFIRI +/− Bevacizumab	Phase III	Ongoing	United States, Argentina, Australia, Austria, Belgium, Brazil, Bulgaria, Canada, Chile, Colombia, Czechia, Denmark, Estonia, France, Germany, Greece, Hungary, Italy, Japan, Mexico, The Netherlands, Poland, Portugal, Romania, South Korea, Spain, Sweden, Switzerland, Taiwan, Thailand, Turkey, United Kingdom
KANDLELIT-012 (NCT06997497) [112]	1st line *KRASG12C* mCRC	MK-1084 + Cetuximab + mFOLFOX6 vs. mFOLFOX6 +/− Bevacizumab	Phase III	Ongoing	United States, Argentina, Australia, Brazil, Canada, China, Colombia, Finland, France, Germany, Hong Kong, Israel, Italy, Japan, The Netherlands, Poland, Romania, Singapore, South Korea, Spain, Taiwan, Ukraine, United Kingdom
MOUNTAINEER-03 (NCT05253651) [135]	1st line HER2 positive mCRC	Tucatinib + Trastuzumab + mFOLFOX6 vs. mFOLFOX6 Given +/− Cetuximab or Bevacizumab	Phase III	Ongoing	United States, Argentina, Australia, Austria, Belgium, Brazil, Canada, Chile, China, France, Germany, Greece, Ireland, Italy, Japan, The Netherlands, Norway, Poland, Portugal, Slovakia, South Korea, Spain, Switzerland, Taiwan, United Kingdom
ZWI-ZW25-201 (NCT03929666) [137]	1st line HER2 positive mCRC	Zanidatamab + mFOLFOX6 +/− Bevacizumab	Phase II	Ongoing (OS not reached)	United States, Canada, Chile, South Korea

Abbreviations: SOC, standard of care.

**Table 3:** Main mechanisms of resistance to targeted therapy in mCRC.

Resistance Mechanism	Molecular Alterations	Category	Affected Pathway(s)	Clinical Relevance
Constitutive pathway activation	*KRAS* and *NRAS* mutations; *BRAFV600E*	Intrinsic	MAPK	Primary resistance to anti-EGFR antibodies
Activation of alternative signaling	*PIK3CA* mutations; *PTEN* loss	Intrinsic	PI3K/AKT/mTOR	Reduced sensitivity to EGFR and MAPK targeted therapies
Receptor amplification	*HER2* amplification	Receptor-level	MAPK, PI3K/AKT/mTOR	Primary and acquired resistance to anti-EGFR therapy
Receptor domain alterations	EGFR extracellular mutations (e.g., S492R, G465R)	Acquired	EGFR	Impaired anti-EGFR antibodies binding
Alternative RTK activation	*MET* amplification; *HER2* overexpression	Bypass signaling	MAPK, PI3K/AKT/mTOR	Resistance to target therapy through activation of downstream signaling
Relief of negative feedback loops	EGFR reactivation after BRAF inhibition	Feedback reactivation	MAPK	Rationale for combined BRAF-EGFR blockade
Restoration of MAPK signaling	KRAS amplification or secondary KRAS mutations	Acquired	MAPK	Resistance to KRAS G12C inhibitors and anti-EGFR therapies
Clonal selection	Expansion of resistant subclones	Tumor heterogeneity	Multiple	Temporary responses and disease progression

Abbreviations: RTK, receptor tyrosine kinase.

## Data Availability

No new data were created or analyzed in this study.

## Abbreviations

The following abbreviations are used in this manuscript:

ADC	Antibody–drug conjugates
ADCC	Antibody-dependent cellular cytotoxicity
ADCP	Antibody-dependent cellular phagocytosis
AF	Allele fraction
APC	Adenomatous polyposis coli
AREG	Amphiregulin
CIN	Chromosomal instability
CMS	Consensus molecular subtypes
CRC	Colorectal cancer
ctDNA	Circulating tumor DNA
mCRC	Metastatic colorectal cancer
dMMR	Mismatch repair-deficient
EGF	Epidermal growth factor
EGFR	Epidermal growth factor receptor
ERBB	Erythroblastosis oncogene B
EREG	Epiregulin
ECD	Extracellular domain
FISH	Fluorescence in situ hybridization
GEFs	Guanine nucleotide exchange factors
HER	Human EGFR
HGF	Hepatocyte growth factor
HIF-1	Hypoxia-inducible factor 1
HIF-2	Hypoxia-inducible factor 2
ICI	Immune-checkpoint inhibitor
IHC	Immunohistochemistry
LGR5	Leucine-rich G-repeat protein
MAF	Mutant allele frequency
MAPK	Mitogen-activated protein kinase
MSI-H	Microsatellite instability–high
MSS	Microsatellite stable
NSCLC	Non-small cell lung cancer
ORR	Objective response rate
OS	Overall survival
PFS	Progression-free survival
PI3K	Phosphoinositide 3-kinase
rMAF	Relative frequencies of mutant alleles
RTK	Receptor tyrosine kinase
SOC	Standard of care
TGFα	Transforming growth factor α
TGF-β	Transforming growth factor-beta
T-DM1	Trastuzumab emtansine
VAF	Variant allele frequency
WT	Wild-type
